# VCAM-1-targeted and PPARδ-agonist-loaded nanomicelles enhanced suppressing effects on apoptosis and migration of oxidized low-density lipoprotein-induced vascular smooth muscle cells

**DOI:** 10.1042/BSR20200559

**Published:** 2020-05-12

**Authors:** Gang Wei, Liangang Hao, Xueli Li, Wen Xu, Fuxiang Liu, Qian Peng, Shoutian Lv

**Affiliations:** Department of Vascular Surgery, Dongying People’s Hospital, 317 Nanyi Road, Dongying District, Dongying City 257091, Shandong Province, China

**Keywords:** Atherosclerosis, Nano micelles, Peroxisome proliferator-activated receptor δ, Vascular cell adhesion molecule-1, Vascular smooth muscle cell

## Abstract

**Purpose:** Nanomicelles (NMs) have been widely used for various biomedical applications due to its unique physiochemical properties. The present study aims to investigate the effects of vascular cell adhesion molecule-1 (VCAM-1)-targeted and peroxisome proliferator-activated receptor δ (PPARδ) agonist (GW0742)-loaded NMs on apoptosis and migration in oxidized low-density lipoprotein (ox-LDL)-induced human aortic vascular smooth muscle cells (HAVSMCs).

**Methods:** The GW0742-loaded NMs (M-GW) and VCAM-1-targeted NMs loaded with GW0742 (TM-GW) were prepared, and then the morphologies and the size distribution of M-GM and TM-GM were observed by transmission electron microscopy (TEM) and dynamic light scattering (DLS), respectively. *In vitro* drug release assay of M-GM and TM-GM were performed as well. Next, HAVSMCs were cultured in medium containing ox-LDL to mimic atherosclerotic environment, and the effects of free GW0742, M-GM and TM-GM on endocytosis, cell migration and apoptosis, as well as the expression of VCAM-1, and proteins associated with migration and apoptosis were measured in HAVSMCs treated with ox-LDL.

**Results:** M-GM and TM-GM were successfully prepared. VCAM-1 was overexpressed in HAVSMCs treated with ox-LDL, and TM-GM had a strong targeting ability to HAVSMCs treated with ox-LDL compared with M-GM. In addition, compared with free GW0742, both M-GM and TM-GM significantly diminished cell apoptosis and migration in HAVSMCs treated with ox-LDL.

**Conclusions:** TM-GM had a superior suppressing effect on apoptosis and migration of ox-LDL-induced HAVSMCs.

## Introduction

As a chronic progressive inflammatory disease, atherosclerosis is one of the most important types of arteriosclerotic vascular diseases, characterized by subendothelial lipoprotein retention and endothelial injury in the arterial wall [1,2]. Atherosclerosis is usually responsible for various cardiac–cerebral vascular disease and peripheral vascular disease, with high rates of disability and mortality [1,3]. Although the drug treatments for atherosclerosis have the intensive progress, unsatisfactory adverse effects such as liver injury and myopathy still exist [4]. Thus, it is essential to search for novel treatment methods of atherosclerosis.

It is well known that the pathogenesis of atherosclerosis is very complex [5]. The initial inflammatory damage of endothelial cells leads to the permeability of lipid-increasing proteins and the adhesion of leukocytes, and then adhere leukocytes to swallow oxidized low-density lipoprotein (ox-LDL) and turn into foam cells. Subsequently, foam cells produce a large number of inflammatory chemokines and increase the adhesion of leukocytes, thereby promoting inflammatory response and the formation of atherosclerotic plaque [5]. Aberrant proliferation, apoptosis and migration of vascular smooth muscle cells (VSMCs) have been proved to be implicated with the occurrence and progression of atherosclerotic plaques despite of the complex pathogenesis [6,7]. VSMCs are the main constituent cells of the blood vessel wall, and play a crucial role in regulating vascular function [8]. Notably, as an adhesion molecule, vascular cell adhesion molecule-1 (VCAM-1) is reported to play critical role in the progression of atherosclerosis by mediating inflammatory response, and it has been considered as an effect target against atherosclerosis [9–11]. Moreover, previous studies also have demonstrated that the activation of peroxisome proliferator-activated receptor δ (PPARδ) can not only inhibit the migration of VSMCs in plaques, but also inhibit apoptosis of VSMCs induced by ox-LDL, which indicate that PPARδ agonist may be a potential treatment for atherosclerosis [12,13].

Currently, nanomicelles (NMs) are reported to be a promising drug delivery system, and widely applied in disease therapy [14]. As the most common nanocarriers, NMs possess several favorable properties (such as high biocompatibility and ability) to carry large drug payloads [14]. Previous studies have developed drug-loaded NMs, which exhibit improved effects compared with free drug [15–17]. Therefore, in the current research, we prepared the VCAM-1-targeted and GW0742 (PPARδ agonist)-loaded NMs, and then characterization and *in vitro* drug release of NMs were detected. In addition, the effects of NMs on apoptosis and migration of VSMCs were unraveled.

## Materials and methods

### Preparation of NMs

Briefly, 2 mg of GW0742 (MedChemExpress, NJ, U.S.A.) was dissolved in 1 ml of absolute methanol, en mixed with 80 μg of Cy5.5 and 10 mg of PEG2000-DSPE that were dissolved in 3 ml of chloroform. Next, the mixture was transferred into the eggplant-shaped bottle. After removing the organic solvent through a vacuum rotary evaporator, a dry lipid film was formed on the bottom of the bottle, and then dissolved in 2 ml of phosphate-buffer saline (PBS) buffer and hydrated in a water bath at 60°C for 30 min. The hydrated solution was filtered through polycarbonate membrane (0.2 μm pore size) to remove unencapsulated hydrophobic molecules, thereby obtaining a GW0742-loaded NMs (M-GW) solution. Similarly, targeted M-GW (TM-GW) were prepared as described above, except that PEG2000-DSPE was replaced by anti-VCAM-1 modified-PEG2000-DSPE (provided by Ruixi Bio Co. Ltd, Xi’an, China).

### Characterization of NMs

The morphological characteristics and particle size parameters of TM-GW were observed by transmission electron microscopy (TEM) and dynamic light scattering (DLS). Briefly, the NMs solution was diluted with deionized water at 0.25 mg/ml with deionized water, and then 10 μl of the sample was dripped on to a carbon-coated copper mesh. After water evaporation, the sample was counterstained with 5 μl of 1% uranyl acetate solution for 30 s, and dried by a 42°C constant temperature dryer. Finally, the sample was observed by TEM (Tecnai G2 20 S-TWIN, FEI, Eindhoven, Netherlands). DLS was performed by Zetasizer Nano Z (Worcestershire, U.K.).

### *In vitro* drug release analysis

The drug release profiles of M-GW and TM-GW at different pH values were analyzed. In brief, 200 μl of M-GW and TM-GW were separately loaded into a dialysis bag (molecular retention of 8000–12000 Da), and the dialysis bag was immersed in 35 ml of PBS buffer (pH 7.4 and 5.0, respectively). PBS buffer with pH 7.4 was used to simulate blood plasma environment and that with pH 5.0 late tumor endocytic compartment. The entire dialysis system was shaken at 200 rpm in a constant temperature shaker at 37°C in the dark. Subsequently, 1 ml of dialysate was taken at 0.5, 1, 2, 4, 6, 9, 12 and 24 h, respectively. Finally, the concentration of GW0742 in the dialysate was determined by fluorescence spectrophotometer, and the *in vitro* release profile was calculated.

### Cell culture and treatment

Human aortic vascular smooth muscle cells (HAVSMCs) were provided by Shanghai Obio (China), and maintained in RPMI-1640 medium (Gibco) with 10% fetal bovine serum (Gibco) under 37°C and 5% CO_2_. Then, HAVSMCs were cultured in medium containing 60 μg/ml of ox-LDL for 24 h to mimic mid- and early-stage atherosclerotic smooth muscle cell migration model, while HAVSMCs were exposed to 150 μg/ml of ox-LDL for 24 h to mimic end-stage atherosclerotic smooth muscle cell apoptosis model. In addition, the expression of VCAM-1 in end-stage atherosclerotic smooth muscle cell apoptosis model was detected by Western blot and cell immunofluorescence. This experiment got the approval of the Medical Ethics Committee of Dongying People’s Hospital and the present study was in-line with the Declaration of Helsinki.

### Cellular uptake of NMs *in vitro*

Cellular uptake of M-GW and TM-GW was evaluated using confocal imaging analysis. Briefly, HAVSMCs (1.5 × 10^5^) were exposed to 60 μg/ml of ox-LDL in 35-mm culture dishes for 24 h, and then incubated with M-GW and TM-GW at 100 nmol/ml of GW0742, respectively, for 1 h. After washing with PBS, cells were observed using confocal fluorescence microscope with 488 nm of excitation wavelength.

### Wound healing assay

HAVSMCs were treated with 60 μg/ml of ox-LDL and/or M-GW/TM-GW at 100 nmol/ml of GW0742. After growing to 60% confluence, cells were wounded by scratching the vertical lineation using pipette tips, followed by culturing in DMEM without serum. Subsequently, cells were treated with 40 and 80 μg/ml GO, respectively. The distance of the scratch was observed at 0 and 12 h using a light microscope (Olympus, Japan).

### Transwell assay

Tumor cell invasion and migration were elevated by Transwell chambers (Corning). Concretely, the bottom compartment was added with 1640 medium supplemented with containing 10% FBS, ox-LDL (60 μg/ml) and/or M-GW/TM-GW (100 nmol/ml of GW0742). HAVSMCs were grown in the upper compartment coated with Matrigel Matrix and cultured in medium free of serum for 24 h. Subsequently, the cells in the bottom compartment were fixed and stained with 4,6-diamidino-2-phenylindole. An inverted microscope (Olympus, Japan) was utilized to evaluate cell migration.

### Cell apoptosis assay

The Acridine Orange/Ethidium Bromide (AO/EB) staining kit and FITC-Annexin V Apoptosis kit were used for the detection of cell apoptosis. HAVSMCs were treated with 60 μg/ml of ox-LDL and/or M-GW/TM-GW at 100 nmol/ml of GW0742 for 24 h. Next, cells were stained with AO/EB for 2 min, and then apoptotic cells with orange fluorescence were observed by fluorescent microscopy. For flow cytometry, cells underwent different treatments were rinsed with PBS, and resuspended with Binding Buffer. After the incubation of FITC-Annexin V and PI with cells for 15 min, flow cytometry (BD, CA, U.S.A.) was used to calculate the number of apoptotic cells.

### Western blot assay

Total proteins from HAVSMCs underwent various treatments were obtained using lysis buffer, respectively, and then quantitated by bicinchoninic acid kit (Beyotime, Shanghai, China). Following sample separation and transfer into PVDF membranes, membranes were immersed in 5% nonfat milk. Next, primary antibodies of VCAM-1, matrix metalloproteinase (MMP) 2 (MMP2), MMP-9, focal adhesion kinase (FAK), P-FAK, TGF-β1, Bim, Bad, Bax, BCl-2, cleaved caspase-3, cleaved caspase-9 (1:800, Abcam) and β-actin (1:1000, Beyotime), respectively, were used for immunoblotting of the membranes overnight at 4°C. After the incubation with secondary antibody (1:5000, Jackson, U.S.A.), the protein concentrations were detected by enhanced chemiluminescence (ECL, Millipore, U.S.A.).

### Cell immunofluorescence

Immunofluorescence assay was carried out as following: briefly, HAVSMCs that were exposed to 150 μg/ml of ox-LDL gradually underwent paraformaldehyde and blockage. Then, the cells were reacted with VCAM-1 antibody (1:50, Abcam) overnight at 4°C, followed by incubation of the FITC–conjugated secondary antibody for 1 h. After washing with PBS, cells were observed by fluorescence microscopy.

### Statistical analysis

Data were presented as the mean ± SD. One-way ANOVA followed by multiple comparison was used for data comparisons based on GraphPad Prism 6 software. *P*<0.05 was considered significant.

## Results

### Characterization and *in vitro* drug release of M-GW and TM-GW

**Figure 1:**
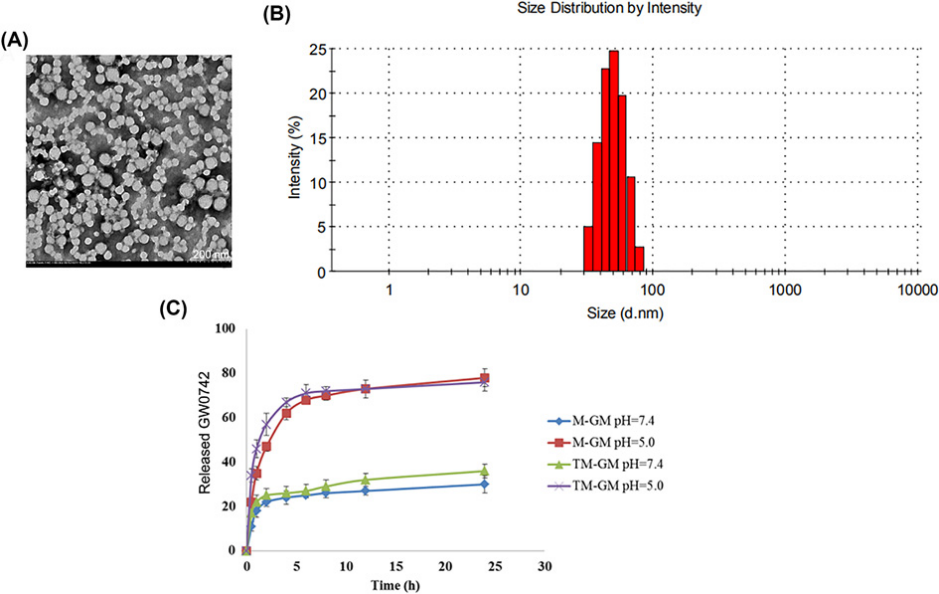
Characterization of TM-GW (**A**) The morphological characteristics of TM-GW observed by TEM. (**B**) Size distributions of TM-GW determined by DLS. (**C**) Cumulative drug release profiles of M-GW and TM-GW in PBS (pH 7.4 and pH 5.0).

As shown in Figure 1A, TEM showed spherical shape of TM-GW with the size of 50–90 nm (Figure 1A). Accordingly, DLS revealed that the hydrodynamic diameter of TM-GW was observed to be approximately 70 nm (Figure 1B). Next, the amounts of GW0742 released from M-GW and TM-GW were examined at pH 7.4 and pH 5.0. Cumulative drug release profiles revealed that both M-GW and TM-GW exhibited a burst release of GW0742 within 5 h and a slow release from 5 to 24 h at pH 7.4 and 5.0 (Figure 1C). Notably, almost 80% of GW0742 was released from both M-GW and TM-GW within 24 h at pH 5.0, which was significantly higher than that at pH 7.4 (Figure 1C).

### Expression of VCAM-1 in end-stage atherosclerotic smooth muscle cell apoptosis model

**Figure 2:**
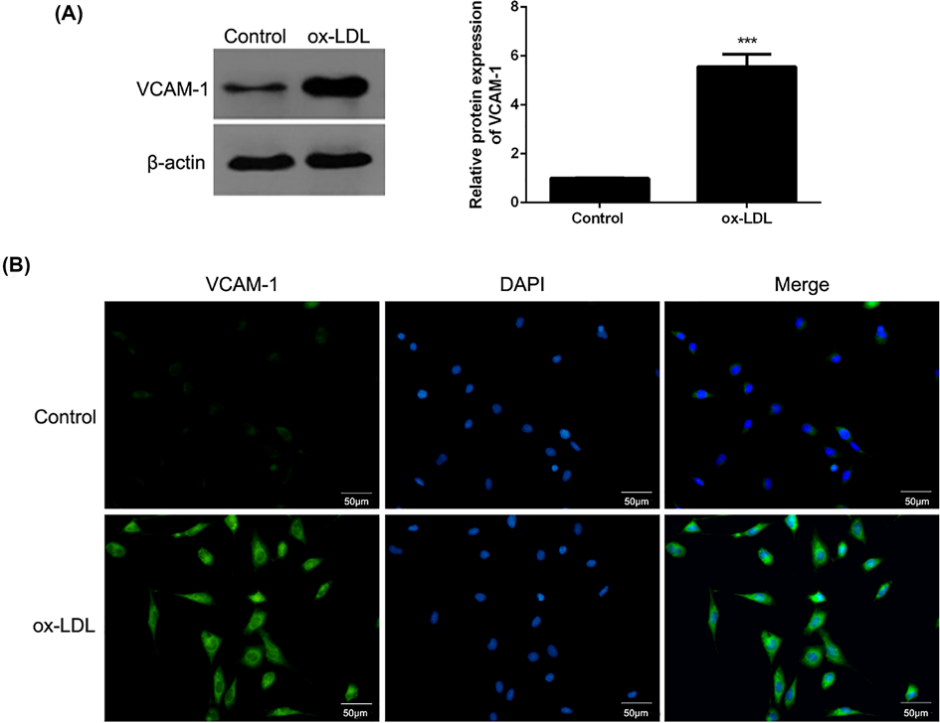
Expression of VCAM-1 in HAVSMCs exposed to 150 μg/ml of ox-LDL (**A**) The protein expression of VCAM-1 in HAVSMCs exposed to 150 μg/ml of ox-LDL by Western blot. (**B**) The expression of VCAM-1 in HAVSMCs exposed to 150 μg/ml of ox-LDL by cell immunofluorescence. ***P<0.001.

The protein expression of VCAM-1 were detected in HAVSMCs that were exposed to 150 μg/ml of ox-LDL (end-stage atherosclerotic smooth muscle cell apoptosis model). The results of Western blot showed that the protein of VCAM-1 was highly expressed in HAVSMCs treated with 150 μg/ml of ox-LDL compared with control cells (*P*<0.001, Figure 2A). Consistently, cell immunofluorescence (Figure 2B) also revealed higher expression of VCAM-1 in HAVSMCs treated with 150 μg/ml of ox-LDL than that in control cells.

### Cellular uptake of M-GW and TM-GW *in vitro*

**Figure 3:**
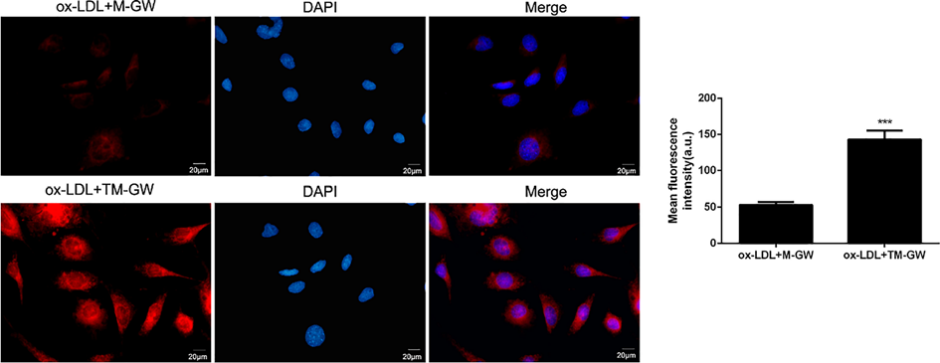
Cellular uptake of M-GW and TM-GW in HAVSMCs Confocal images of ox-LDL (60 μg/ml)-induced HAVSMCs treated with M-GW and TM-GW at 100 nmol/ml of GW0742 for 1 h. ***P<0.001.

Based on confocal imaging analysis, we found the enhanced fluorescence intensity when ox-LDL (60 μg/ml)-induced cells were treated with TM-GW compared with M-GW (*P*<0.001, Figure 3), which indicated that the addition of anti-VCAM-1 increased the cellular uptake of NMs.

### *In vitro* anti-migration effect of M-GW and TM-GW

**Figure 4:**
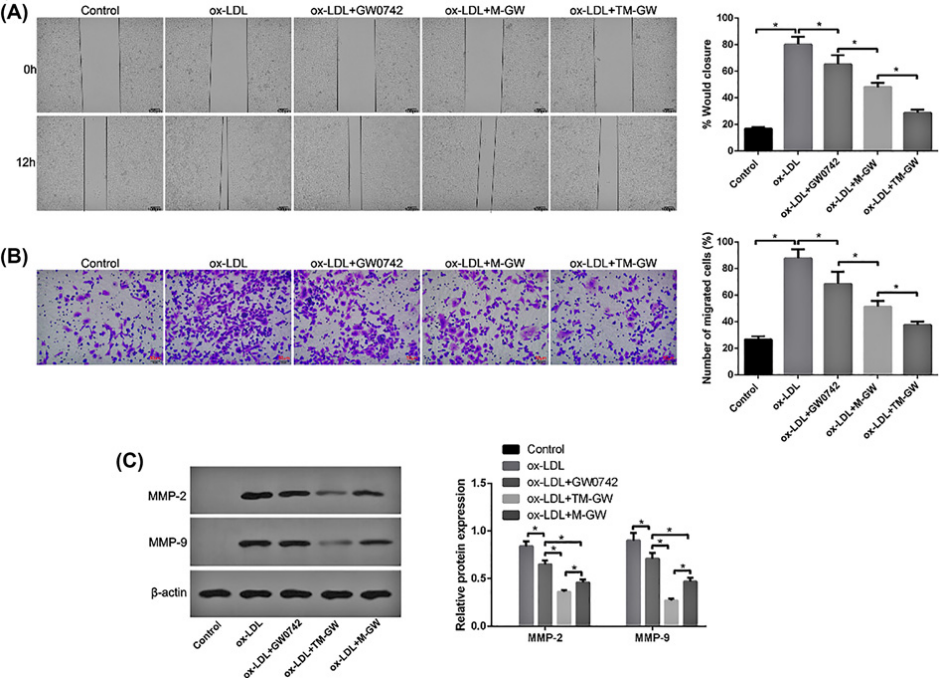
TM-GW inhibited cell migration in ox-LDL (60 μg/ml)-treated HAVSMCs (**A**) The wound closure rate of HAVSMCs treated with PBS (control), ox-LDL, ox-LDL + GW0742, ox-LDL + M-GW, or ox-LDL + TM-GW at 12 h by wound healing assay. (**B**) Cell migration rate of HAVSMCs treated with PBS (control), ox-LDL, ox-LDL + GW0742, ox-LDL + M-GW, or ox-LDL + TM-GW by Transwell assay. (**C**) The expression of metastasis-related proteins, including MMP2 and MMP9, in HAVSMCs treated with PBS (control), ox-LDL, ox-LDL + GW0742, ox-LDL + M-GW, or ox-LDL + TM-GW by Western blot. *P<0.05.

Wound healing assay showed that 60 μg/ml of ox-LDL treatment significantly increased the wound closure of HAVSMCs compared with control cells (*P*<0.05, Figure 4A), suggesting the successful establishment of mid- and early-stage atherosclerotic smooth muscle cell migration model. However, the addition of free GW0742 reduced the wound closure of HAVSMCs compared with cells treated with ox-LDL (*P*<0.05, Figure 4A). In addition, the co-treatment of ox-LDL and M-GW or TM-GW further inhibited the wound closure of HAVSMCs compared with cells treated with ox-LDL and free GW0742, especially TM-GW treatment (*P*<0.05, Figure 4A). Similarly, Transwell assay also revealed that cell migration rate was dramatically increased in HAVSMCs treated with ox-LDL compared with control cells (*P*<0.05, Figure 4B), while cell migration rate was obviously decreased after the addition of free GW0742, M-GW or TM-GW (*P*<0.05, Figure 4B). The highest cell migration rate was found in HAVSMCs treated with ox-LDL and TM-GW, followed by M-GW and free GW0742 (*P*<0.05, Figure 4B). Meanwhile, the expressions of metastasis-related proteins, including MMP2 and MMP9, were detected by Western blot. The results found that ox-LDL treatment remarkably increased the protein concentrations of MMP2 and MMP9 compared with control cells (*P*<0.05, Figure 4C). Moreover, compared with cells treated with ox-LDL, the expression of MMP2 and MMP9 was obviously decreased after the addition of free GW0742, M-GW or TM-GW (*P*<0.05, Figure 4C), with the highest difference in HAVSMCs treated with ox-LDL and TM-GW, followed by M-GW and free GW0742 (*P*<0.05, Figure 4C).

### *In vitro* anti-apoptosis effect of M-GW and TM-GW

**Figure 5:**
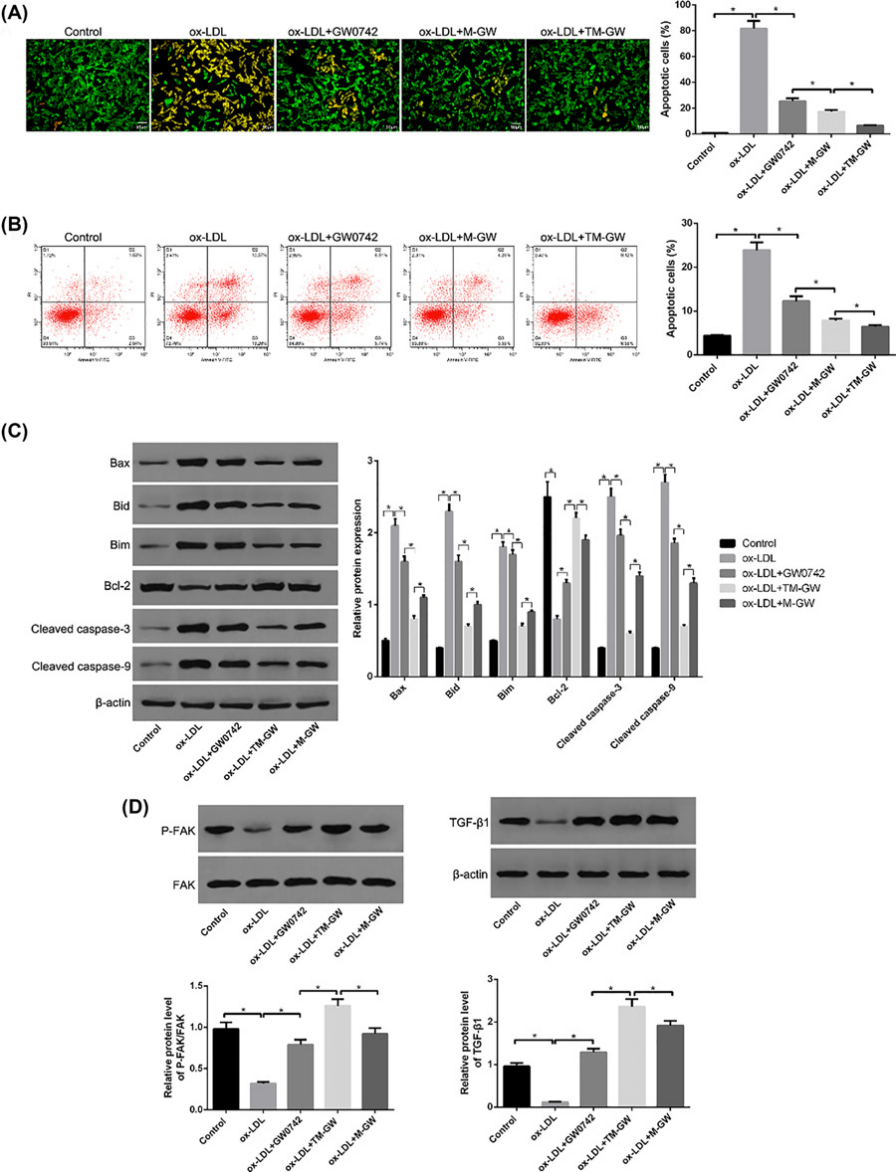
TM-GW inhibited cell apoptosis in ox-LDL (60 μg/ml)-treated HAVSMCs (**A**) Cell apoptosis rate of HAVSMCs treated with PBS (control), ox-LDL, ox-LDL + GW0742, ox-LDL + M-GW, or ox-LDL + TM-GW using AO/EB staining kit. (**B**) Cell apoptosis rate of HAVSMCs treated with PBS (control), ox-LDL, ox-LDL + GW0742, ox-LDL + M-GW, or ox-LDL + TM-GW by flow cytometry analysis. (**C**) The expression of apoptosis-related proteins, including Bim, Bad, Bax, BCl-2, cleaved caspase-3, and cleaved caspase-9, in HAVSMCs treated with PBS (control), ox-LDL, ox-LDL + GW0742, ox-LDL + M-GW, or ox-LDL + TM-GW by Western blot. (**D**) The expression of FAK, P-FAK, and transforming growth factor (TGF)-β1 in HAVSMCs treated with PBS (control), ox-LDL, ox-LDL + GW0742, ox-LDL + M-GW, or ox-LDL + TM-GW by Western blot. *P<0.05.

AO/EB staining assay showed that 150 μg/ml of ox-LDL treatment significantly increased the rate of apoptotic cells compared with control cells (*P*<0.05, Figure 5A), suggesting the successful establishment of end-stage atherosclerotic smooth muscle cell apoptosis model. However, the addition of free GW0742 significantly decreased the rate of apoptotic cells compared with cells treated with ox-LDL; meanwhile, compared with cells with free GW0742, cells co-treated with ox-LDL and M-GW showed the decreased rate of apoptotic cells, and then co-treatment of ox-LDL and M-GW further reduced the rate of apoptotic cells (*P*<0.05, Figure 5A). Moreover, the flow cytometry analysis revealed the consistent results with AO/EB staining assay (*P*<0.05, Figure 5B). In addition, the expressions of apoptosis-related proteins, including Bim, Bad, Bax, BCl-2, cleaved caspase-3, cleaved caspase-9, were detected by Western blot. The results found that ox-LDL treatment remarkably increased the protein concentrations of Bim, Bad, Bax, cleaved caspase-3 and cleaved caspase-9, while decreased the protein concentration of Bcl-2 compared with control cells (*P*<0.05, Figure 5C). Moreover, compared with cells treated with ox-LDL, the expressions of Bim, Bad, Bax, cleaved caspase-3 and cleaved caspase-9 were obviously decreased, while the expression of Bcl-2 was remarkably elevated after the addition of free GW0742, M-GW or TM-GW (*P*<0.05, Figure 5C), with the highest difference in HAVSMCs treated with ox-LDL and TM-GW, followed by M-GW and free GW0742 (*P*<0.05, Figure 5C). Furthermore, the expressions of P-FAK and TGF-β1 were detected, and the results showed decreased protein concentrations of P-FAK and TGF-β1 in ox-LDL-treated HAVSMCs compared with control cells, and increased concentrations of P-FAK and TGF-β1 in ox-LDL-induced HAVSMCs treated with free GW0742, M-GW and TM-GW, in turn (*P*<0.05, Figure 5D).

## Discussion

It is well known that VSMCs play important roles in the formation and progression of atherosclerotic plaques, and increasing studies have reported that targeting VSMCs is suggested to be a critical strategy to alleviate atherosclerosis [6]. Thus, in the present study, we investigated the effect of PPARδ agonist GW0742 on ox-LDL-induced HAVSMCs. PPARδ as a member of PPAR family has been considered to exert important roles in cellular inflammatory responses and lipid homeostasis [18]. Interestingly, several studies have demonstrated that PPARδ agonists such as GW0742 and GW1516 can prevent the progression of atherosclerosis through regulating inflammatory response [19–21]. However, due to poor water solubility, GW0742 may be clinically limited to apply into disease treatment. NMs have been reported to be a promising drug delivery system to overcome the application limitation of hydrophobic drugs because of its attractive properties, including highly hydrophilic nature and excellent biocompatibility [22,23]. Thus, the present study developed M-GWs, and evaluated the effect of M-GWs on ox-LDL-induced HAVSMCs.

The present study revealed that ox-LDL treatment significantly increased cell migration and apoptosis of HAVSMCs; however, free GW0742 treatment inhibited cell migration and apoptosis in ox-LDL-induced HAVSMCs, and then M-GW further diminished cell migration and apoptosis in ox-LDL-induced HAVSMCs compared with free GW0742. In addition, the present study detected the expression of MMP2 and MMP9, and the results found that M-GW remarkably decreased the protein concentrations of MMP2 and MMP9. Both MMP-2 and MMP-9 are important members of the MMP family, and are also implicated with cell migration by medicating inflammatory cell infiltration in atherosclerosis [24]. Moreover, the concentrations of apoptosis-related proteins, including Bim, Bad, Bax, Bcl-2, cleaved caspase-3, cleaved caspase-9, were measured. Bim, Bad, Bax and Bcl-2 belong to Bcl-2 protein family, which exert significant role in cell apoptosis, and Bim, Bad and Bax are pro-apoptosis proteins, while Bcl-2 is anti-apoptosis protein [25]. It is well known that Bcl-2 protein family is closely associated with mitochondria-mediated apoptosis pathway [26]. As a downstream molecule of apoptosis pathway, caspase-3 can inhibit the ratio of Bcl-2/Bax and then contribute to cell apoptosis [26]. In addition, caspase-3 also can be activated followed by the recruitment and activation of caspase-9 [27]. Notably, we found that ox-LDL treatment significantly decreased protein concentrations of P-FAK and TGF-β1 in HAVSMCs; however, free GW0742 treatment increased their expression in ox-LDL-induced HAVSMCs, and then M-GW further promoted the expression of P-FAK and TGF-β1 in ox-LDL-induced HAVSMCs compared with free GW0742. Both the activations of FAK and TGF-β1 have been proved to be implicated in cell apoptosis and migration [28,29]. Thus, we speculated that FAK/TGF-β1 signaling pathway participated in the effects of M-GW on cell apoptosis and migration in ox-LDL-induced HAVSMCs.

Furthermore, to enhance the accuracy of M-GW targeting to HAVSMCs, anti-VCAM-1 modified M-GW (TM-GW) was prepared. The presentstudy found that VCAM-1 was over-xpressed in ox-LDL-induced HAVSMCs, and TM-GW exhibited increased the cellular uptake than M-GW. Previous study has demonstrated that targeting VCAM-1 with nanoparticles can be effectively utilized in the treatment of atherosclerosis [30]. Similarly, the present study revealed that TM-GW exhibited enhanced inhibiting effects on cell apoptosis and migration compared with M-GW in ox-LDL-induced HAVSMCs.

In conclusion, the present study successfully developed that M-GW and TM-GW, and TM-GW hL by inhibiting VCAM-1 and PPARδ. Overall, TM-GW might be a promising therapeutic nanomedicine in atherosclerosis; however, the further *in vivo* experiments should be investigated.

## Abbreviations

AO/EB: Acridine Orange/Ethidium Bromide
Bad: BCL2 associated agonist of cell death
Bax: BCL2 associated X, apoptosis regulator
BCL2: BCL2 apoptosis regulator
Bim: BCL2 like 11
DLS: dynamic light scattering
FAK: focal adhesion kinase
GO: Gene Ontology
HAVSMC: human aortic vascular smooth muscle cell
MMP: matrix metalloproteinase
M-GW: GW0742-loaded nanomicelle
NM: nanomicelle
ox-LDL: oxidized low-density lipoprotein
PBS: phosphate-buffer saline
PI: Propidium lodide
PPARδ: peroxisome proliferator-activated receptor δ
TEM: transmission electron microscopy
TM-GW: targeted M-GW
VCAM-1: vascular cell adhesion molecule-1
