# Expression of Concern: VCAM-1-targeted and PPARδ-agonist-loaded nano micelles enhanced suppressing effects on apoptosis and migration of oxidized low-density lipoprotein-induced vascular smooth muscle cells

**DOI:** 10.1042/BSR-20200559_EOC

**Published:** 2020-06-24

**Keywords:** nano micelles, vascular cell adhesion molecule-1, peroxisome proliferator-activated receptor δ, atherosclerosis, vascular smooth muscle cell

The Editorial Office has been made aware of potential issues surrounding the scientific validity of this paper, hence has issued an expression of concern to notify readers whilst the Editorial Office investigates.

